# Biomarkers of oxidative stress and its association with the urinary reducing capacity in bus maintenance workers

**DOI:** 10.1186/1745-6673-6-18

**Published:** 2011-05-30

**Authors:** Jean-Jacques Sauvain, Ari Setyan, Pascal Wild, Philippe Tacchini, Grégoire Lagger, Ferdinand Storti, Simon Deslarzes, Michel Guillemin, Michel J Rossi, Michael Riediker

**Affiliations:** 1 Institute for Work and Health, University of Lausanne + Geneva, 21 rue du Bugnon, CH-1011 Lausanne, Switzerland; 2 EDEL Therapeutics S.A., PSE-B/EPFL, CH-1015 Lausanne, Switzerland; 3 Paul Scherrer Institute, Laboratory of Atmospheric Chemistry (LAC), CH-5232 Villigen PSI, Switzerland; 4 University of California, Davis; Department of Environmental Toxicology, 4422 Meyer Hall, One Shields Avenue, Davis CA 95616 USA

## Abstract

**Background:**

Exposure to particles (PM) induces adverse health effects (cancer, cardiovascular and pulmonary diseases). A key-role in these adverse effects seems to be played by oxidative stress, which is an excess of reactive oxygen species relative to the amount of reducing species (including antioxidants), the first line of defense against reactive oxygen species. The aim of this study was to document the oxidative stress caused by exposure to respirable particles *in vivo*, and to test whether exposed workers presented changes in their urinary levels for reducing species.

**Methods:**

Bus depot workers (n = 32) exposed to particles and pollutants (respirable PM_4_, organic and elemental carbon, particulate metal content, polycyclic aromatic hydrocarbons, NO_x_, O_3_) were surveyed over two consecutive days. We collected urine samples before and after each shift, and quantified an oxidative stress biomarker (8-hydroxy-2'-deoxyguanosine), the reducing capacity and a biomarker of PAH exposure (1-hydroxypyrene). We used a linear mixed model to test for associations between the oxidative stress status of the workers and their particle exposure as well as with their urinary level of reducing species.

**Results:**

Workers were exposed to low levels of respirable PM_4 _(range 25-71 μg/m^3^). However, urinary levels of 8-hydroxy-2'-deoxyguanosine increased significantly within each shift and between both days for non-smokers. The between-day increase was significantly correlated (p < 0.001) with the concentrations of organic carbon, NO_x_, and the particulate copper content. The within-shift increase in 8OHdG was highly correlated to an increase of the urinary reducing capacity (Spearman ρ = 0.59, p < 0.0001).

**Conclusions:**

These findings confirm that exposure to components associated to respirable particulate matter causes a systemic oxidative stress, as measured with the urinary 8OHdG. The strong association observed between urinary 8OHdG with the reducing capacity is suggestive of protective or other mechanisms, including circadian effects. Additional investigations should be performed to understand these observations.

## Background

Epidemiological studies have demonstrated that increased levels of airborne particles are associated with adverse health effects, such as cancer, cardiovascular and pulmonary diseases [1]. Among the different mechanisms proposed to explain these adverse effects, the production of reactive oxygen species (ROS) and the generation of oxidative stress have received most of the attention. ROS include both oxygenated radicals and certain closed shell species that are oxidizing agents. Under normal coupling conditions in the mitochondrion, ROS are generated at low frequency and are easily neutralized by antioxidant defenses. However, in the presence of oxidants, such as following exposure to particles, the natural antioxidant defenses may be overwhelmed [2]. Oxidative stress refers to an imbalance between pro-oxidant and antioxidant in favor of the former, leading to potential damage. The biological effect of ROS depends on its local concentration. When the local levels are high, they tend to react with biological structures (DNA, cell membranes and others) leading to cell damage as well as the generation of other reactive radicals. At lower concentrations, however, some ROS can become a secondary messenger, modulating the expression of signaling molecules or proteins (redox signaling function) [3]. In the lungs, rapid build-up of oxidative stress in the thin liquid layer of the alveolar region has been suggested as a consequence of particle deposition. It leads to epithelial cell damage and to the release of pro-inflammatory mediators [4].

Diesel particles are complex objects consisting of a solid carbonaceous core on which many organic, persistent free radicals, inorganic, and metallic compounds are adsorbed. Among these, polycyclic aromatic hydrocarbons (PAHs) [5] and transition metals [6] have been found to cause oxidative stress. A three-tier hierarchical cellular response model has been proposed [7] to explain the role of oxidative stress in mediating its biological effects. This model suggests that low levels of oxidative stress induce protective effects (tier-1) by the activation of antioxidant enzymes. If these responses fail to provide adequate protection, then a further increase in ROS production will result in pro-inflammatory (tier-2) and cytotoxic (tier-3) effects. Taken together, this model expands the above described mechanism to understand how particles generate adverse health effects.

Over the past 15 years, urinary 8-hydroxy-2'-deoxyguanosine (8OHdG) has been widely used as a biomarker of oxidative DNA damage in air pollutant studies. Exposure to diesel [8] and fine particles [9-13], PAHs [14] or metals [9,15-17] were found to significantly increase urinary levels of 8OHdG. Two recent meta-analysis proposes urinary 8OHdG to be a suitable biomarker for evaluating the effect of exposure to PM on humans [18,19]. Such a biomarker would have a predictive value regarding the development of lung cancer [19]. A steady state pool of oxidized nucleobases is considered to be maintained at a cellular level and the urinary excretion of 8OHdG can be considered as a measure of the whole-body oxidative stress [20-22]. The presence of 8OHdG in urine seems to originate mostly from the oxidation of the deoxynucleotide pool [19,23] and does not represent solely repairing/excretion of the oxidized-DNA guanine. Once produced, 8OHdG is very stable and is not further metabolized in the systemic circulation [23]. After exposure to oxidants, the repair and final 8OHdG excretion in urine is rapid, i.e. within at least 24 hours [19,24,25].

The aims of this study were to test *in vivo *whether exposure to particles was associated to oxidative stress and, as indicator for an adaptive response, if an increase of the systemic anti-oxidant defenses could also be detected in urine. For that purpose, we conducted an occupational field study at three bus depots where we expected workers to be exposed to high levels of respirable particles. We assessed worker's exposure to respirable particles with aerodynamic diameter smaller than 4 μm (PM_4_), organic carbon (OC), elemental carbon (EC), three metals (Fe, Cu, Mn) and some particle-bound PAHs. We also collected spot urine samples to quantify in it 8OHdG, the global amount of reducing species, and a biomarker of PAH exposure (1-hydroxypyrene [1-OHP]). The first tier of the defense mechanism against oxidative stress [7] was verified by testing the correlation between levels of 8OHdG, reflecting oxidative stress, and the reducing capacity (corresponding to a defense against oxidative stress) in the urine of the particle-exposed workers.

## Methods

### Subjects and study design

Participating workers (n = 32) were recruited in three bus depots in southwestern Switzerland. The main task of these workers was the repair and maintenance of buses. They were exposed to diesel particles as well as other particles and organic compounds (solvents, diesel fuels, lubricating oil, cigarette smoke). Stationary and personal air sampling were conducted in each bus depot for two consecutive days of shift, between Monday morning and Tuesday evening. Workers did not work the two days preceeding the study. This study design was chosen in order to obtain a large exposure contrast. For that reason, we followed the workers during day and night shifts as well as during summer and winter time. We used a panel study design a) to determine the temporal changes of urinary biomarkers for the participating workers during two consecutive days and b) to use each worker as its own control by considering the Monday morning as the reference value for all biological end-points. This design excluded confounding factors that are stable within an individual over time but vary between participants. The study was approved by the Ethics Committee of the University of Lausanne. Written informed consent was obtained prior to start of the study, in addition to questionnaires destined to collect information on possible confounding factors (cigarette smoke, eating habits, diseases, medication).

### Exposure characterization

The respirable fraction reaching the alveolar region of the lungs was determined by measuring PM_4_, the reference metric for alveolar dust at the workplace [26] (note that this is different from ambient situations, where PM_2.5 _is considered to be the reference). These concentrations were determined either with stationary or personal sampling devices. The stationary sampling was located indoor as close as possible to the worker's place. It consisted of two high-volume pumps (Digitel, model DH 77, 580 L/min with PM_4 _impactor), equipped with passivated 15 cm Whatman QM-A quartz filters as previously described [27]. The personal pumps, connected to a cyclone head were run at a flow of 2 L/min during the entire shift. Plasma pre-treated quartz filters (Whatman QM-A, 37 mm, 2.2 μm pore size) were conditioned at least 24 hours at constant humidity (60 ± 10%) and ambient temperature before weighing. After the sampling, filters were conditioned again and weighed. The limit of detection was 10 μg/m^3^. For comparison, two personal pumps with the same collection head and filters were collocated with the stationary high-volume pumps. All these gravimetric measurement methods were accredited following the ISO/IEC 17025 norm.

The determination of the OC and EC content of particles was carried out on the same filters used for the PM_4 _determination (personal and stationary pumps). The measurement [28] was performed with a Stroehlein Instrument, model 702, and consisted of a coulometric determination of the CO_2 _evolved from a two-stage thermal decomposition of the carbonaceous compounds present in the particles. The OC content refers to the amount of carbon evolved up until 800°C under a stream of nitrogen, whereas the EC content is measured by heating the residue at 800°C under oxygen. The detection limit was 3 μg/m^3 ^for OC and 2 μg/m^3 ^for EC. The analytical method was accredited following the ISO/IEC 17025 norm.

As iron (Fe), copper (Cu) and manganese (Mn) may be involved in ROS production such as the Fenton reaction, we have determined its levels on the PM_4 _samples collected by the high-volume sampler. Five punches (48 mm diameter) were cut and used for the metal analysis. The rest of the filter was used for subsequent PAH analysis. After digestion in hydrogen fluoride followed by a treatment in aqua regia (HNO_3_:HCl 1:2 v/v) and dilution in water, the metal content of the resulting solution was analyzed using an atomic absorption spectrometer (Perkin Elmer, model HGA 700). Results obtained for each sample were corrected by subtraction of a blank filter. The detection limits were 7, 3.5, and 2 ng/m^3 ^for Fe, Cu, and Mn, respectively. The analytical method was accredited following the ISO/IEC 17025 norm.

As workers in this study are exposed to combustion related compounds, PAH adsorbed on particles were expected to be present at these working conditions. As mentioned before, the rest of the high-volume filter was used for PAH analysis. Six semi-volatile PAH (Benzo[a]Anthracene, Benzo[b+j]Fluoranthene, Benzo[k]Fluoranthene, Benzo[a]Pyrene (B[a]P), Indeno[1,2,3-cd]Pyrene, Dibenz[a,h]Anthracene) were determined by gas chromatography-mass spectrometry (GC-MS), as described in reference [29]. The limit of detection for each PAH, based on three times the noise, was 0.002 ng/m^3^. As the recovery of the selected PAH was higher than 90%, the concentrations were not corrected for loss during analysis. The final results were expressed as B[a]P equivalent (B[a]P_eq_), by using the potency equivalent factor of each individual compound as previously described [30].

Gaseous oxidants like NO_2 _or NO are present in diesel exhaust emissions, whereas O_3 _is another common oxidant gas found in the atmosphere. Direct reading instruments were used to monitor the concentrations of NO_x _(Monitor Labs Inc, model ML 9841A) and ozone (Monitor Labs Inc, model ML 9810). These instruments were located next to the stationary high-volume samplers. For the calibration of the NO_x _analyzer, we diluted 40 ppm NO (Carbagas, Gümligen; mixture 40 ppm NO 30, balance N_2 _60, 10 L, 150 bar) with air (Carbagas; controlled air, 30 L, 200 bar) to obtain the following NO concentrations: 0 (zero air: controlled air cleaned through two tubes filled with activated charcoal and a third one filled with silicagel), 250, 500, 750, 1000 ppb. For the calibration of the ozone analyzer, we used an ozone generator (Horiba Ltd). The calibration was achieved with the following ozone concentrations: 0 (zero air), 25, 50, 75, 100 ppb. The limit of detection was 0.5 ppb for the NO_x _as well as for ozone.

### Urine sample collection

Spot urine samples of workers were collected before and after shifts on Monday and Tuesday in pre-cleaned plastic bottles. Urine samples were stored at 4°C in the bus depots and, at the end of the sampling day, were transferred to storage at -25°C in the dark until analysis. In such conditions, the 8OHdG and 1-OHP stability are 15 years [23] and at least 6 months [31], respectively.

### Measurement of 1-OHP in urine

The analysis of 1-OHP, a metabolite of pyrene, is proposed as a reliable biomarker of the internal dose for PAH exposure [32]. However, it is not representative of genotoxic PAH exposure, as pyrene is not a carcinogenic compound [33]. The urinary 1-OHP was analyzed following an ISO/EN17025 accredited method. Briefly, the sample was first digested with glucoronidase at 37°C for at least 2 hours. The hydrolysate was loaded on a C_18 _SPE cartridge, pre-conditioned with methanol and water. After lavage with 4 mL water and 2 mL hexane, the analyte was eluted with 3.5 mL dichloromethane. The extract was concentrated to about 200 μL and injected into a HPLC system equipped with fluorescence detection. The detection limit was 0.01 μg/L. Internal quality control was introduced during each series and obtained using a doped stock urine, whose mean concentration was 1.49 ± 0.14 μg/L (n = 27). The mean value of the internal controls was 1.52 ± 0.05 μg/L (n = 5).

### Measurement of 8OHdG in urine

The analysis of 8OHdG was performed using liquid chromatography-tandem mass spectrometry (LC-MS/MS), preceded by a clean-up procedure with solid phase extraction (SPE). The analytical method was taken from a previously published clean-up procedure [34] and adapted to the conditions of analysis by LC-MS/MS [35]. Prior to the analyses, the urine samples were thawed, and 1.5 mL urine was mixed with an equal volume of bidistilled water. If the urine pH was higher than 7.0, samples were acidified with 20 μL of HCl 2 M. BondElut C_18_/OH SPE cartridges (500 mg, 3 mL, BioPack Switzerland) and were conditioned using 4 mL methanol and 4 mL bidistilled water, then loaded with 2 ml of diluted urine sample, and washed with 4 mL bidistilled water and 4 mL methanol 5% in bidistilled water. 8OHdG was eluted with 7 mL methanol 15% in bidistilled water, and concentrated up to approximately 1 mL in a SpeedVac concentrator (model SVC 100 H, Savant Instruments Inc.). The final volume was determined by gravimetry, assuming that the entire methanol was removed during the concentration in the SpeedVac and that the density of the remaining solvent is 1 g/mL. 20 μL of the samples were injected into a LC-MS/MS system (Varian Inc, model 1200L) equipped with a Polaris C_18_-A analytical column (Varian Inc; length = 50 mm, inner diameter = 2 mm, porosity 5 μm). The parameter settings of the LC-MS/MS are given in the Additional file 1 Table S1. 8OHdG was identified on the chromatograms by the retention time (2.4 min), and quantified by using an eight-point calibration curve in the concentration range 0.9-175.2 pg/μL. The detection limit (based on three times the noise) and the recovery rate for urine samples were 1.04 ± 0.39 μg/L (3.67 ± 1.39 nM) (n = 5) and 73 ± 12% (n = 5), respectively. Urinary concentrations of 8OHdG were ratioed to creatinine for normalization, and the results expressed in terms of μg 8OHdG/g creatinine. The creatinine concentration was determined following the Jaffe method. In the case of repeated measurement of the same individual, there is an acceptable association between the 8OHdG concentration in the creatinine-corrected spot urine and the 24 hour urine [24]. Thus, the creatinine correction may be applied in the present case.

### Measurement of the reducing capacity in urine

We used a novel redox sensor to measure the levels of reducing species in the urine samples. This technique is an electrochemical-based method responding to all water soluble compounds in biological fluids (saliva, serum, urine) which can be oxidized within a defined potential range [36,37]. This assay has been shown to respond linearly to low molecular weight antioxidants like ascorbic and uric acid (P. Tacchini, personal communication). The non-specificity of this assay is an advantage in the present case, because we primarily wanted to detect whether a systemic defense mechanism was taking place after exposure to oxidants like diesel particles. A minimum volume of 10 μl of sample was loaded onto a chip, and an increasing potential between 0 and +1.2 V (vs Ag/AgCl reference electrode) was applied between two carbon based printed conductors. For each compound undergoing an oxidation reaction within this range of potential, a proportional contribution to the current was recorded. Since the potential was increasing from low to high voltage, only compounds in their reduced state will be measured using such a method. Results are expressed in μW/g creatinine. The factors controlling dilution of a urinary reducing compound will also control the concentrations of normal constituents of urine, if they are excreted by the same mechanisms. The electrochemical measurement detects the presence of compounds like uric acid and a close association between the 24 hour excretion of creatinine and uric acid has been reported [38] justifying the creatinine normalization in this study. The detection limit was 13 μW/g creatinine. As 8OHdG is also an electro-active compound, we verified that the levels present in the urine did not interfere with this measurement.

### Statistical analyses

Statistical analyses were performed using Stata 10 (College Station, Tx). Urinary concentrations of 1-OHP, 8OHdG and reducing capacity were log-transformed to normalize their distribution. The evolution of log(1-OHP), log(8OHdG) and log(reducing capacity) was analyzed using a linear mixed model with the subject considered as a random effect and considering within-day and between-day differences as main independent effects. A fixed effect model was also applied to check the robustness of the results. Adjustments were applied when statistically significant differences were found for season, night vs. day shift, body mass index (BMI), self-declared exposure during the preceding week-end, self-reported respiratory diseases and current smoking. Interactions were explored between smoking status and the between- and within-day differences. Residual plots allowed the identification of potential outliers, which were tentatively excluded in subsequent analyses to assess the robustness of the results.

## Results

### Description of the studied subjects and sampling sites

The characteristics of the recruited workers, all male mechanics from three bus depots in Switzerland, are given in Table 1. Twenty-three workers were non-smokers or former smokers (smoking stopped for an average of 13 years, minimum of 2 years), and nine were smokers. None of the workers was excluded. Eight workers reported allergies (4 non-smokers and 4 smokers), two heart problems, and nine used medications (5 non-smoker and 4 smokers), including vitamin/mineral supplements. This information was included in the mixed models. The different sampling sites were large yards (between 70-140'000 m^3^) used as vehicle depot and for mechanical repair and vehicle maintenance (see Additional file 1 Table S2).

**Table 1:** Characteristics of the studied male workers.

	All subjects	Non-smoker	Smoker
Number of workers	32	23	9
Age, year (mean ± SD)	43.1 ± 9.3	43.0 ± 9.0	43.3 ± 10.8
BMI, kg/m^2 ^(mean ± SD)	25.2 ± 3.6	25.6 ± 3.2	24.2 ± 4.5
Years of employment (mean ± SD)	11.8 ± 9.2	11.5 ± 9.1	12.7 ± 9.7

Characteristics of the studied male workers.

### Occupational exposure to particles and pollutants

Table 2 shows the mean stationary and personal concentrations of particles and pollutants measured during the investigated shifts. Stationary PM_4 _concentrations were between 43 and 71 μg/m^3 ^during daytime, and between 25 and 32 μg/m^3 ^during the nighttime shift. OC concentrations ranged from 16-35 μg/m^3 ^and EC concentrations from 6-16 μg/m^3^. PM_4 _and OC were strongly correlated (r^2^: 0.94; Pearson < 0.001). Metal concentrations varied strongly across the sampling site. The sequence of metal concentrations was usually Fe > Cu > Mn, except for bus depot 3 in summer, where the particulate manganese content was higher than that of copper. B[a]P equivalent concentrations (B[a]P_eq_) ranged from 0.17 to 9.56 ng/m^3^. NO_x _levels were between 190 and 920 ppb, and very variable depending on the sampling site. Ozone concentrations were negligibly low, as expected (range 1 to 13 ppb).

**Table 2:** Stationary and personal concentrations of particles and gaseous pollutants measured at the different workplaces during two consecutive days of an 8-hour period of shift (day or night shift as indicated).

Parameter	Depot 1 day	Depot 2 day	Depot 2 night	Depot 2^a^ day	Depot 3 day	Depot 3 night
**Stationary measurements^b^**						
PM_4 _[μg/m^3^]	71 ± 11	52 ± 2	32 ± 15	59 ± 12	43 ± 3	25 ± 9
OC [μg/m^3^]	29 ± 2	24 ± 2	30 ± 4	35 ± 4	26 ± 0	16 ± 6
EC [μg/m^3^]	16 ± 1	7 ± 1	7 ± 1	7 ± 2	7 ± 1	6
Fe [ng/m^3^]	1280 ± 173	2346 ± 292	1053 ± 679	2907 ± 1213	323 ± 100	1459 ± 1454
Cu [ng/m^3^]	105 ± 51	48 ± 23	17 ± 5	186 ± 53	12 ± 2	75 ± 86
Mn [ng/m^3^]	9 ± 3	27 ± 1	13 ± 8	29 ± 1	25 ± 33	13 ± 14
B[a]P_eq _[ng/m^3^]	9.6 ± 0.8	0.85 ± 0.2	0.40 ± 0.3	1.6 ± 0.2	1.1 ± 0.1	0.2 ± 0.1
NO [ppb]	431 ± 69	n.a^c^	n.a	781 ± 99	445 ± 218	176 ± 98
NO_2 _[ppb]	117 ± 11	n.a	n.a	136 ± 13	31 ± 16	17 ± 9
NO_x _[ppb]	547 ± 79	n.a	n.a	917 ± 112	476 ± 234	192 ± 107
O_3 _[ppb]	1.4 ± 0.2	2.3 ± 0.1	4.3 ± 0.8	1.7 ± 0.9	4.3 ± 4.7	12.9 ± 4.9

**Personal measurements^d^**						
PM_4 _Non smoker	99 ± 49 (12)	73 ± 50 (6)	125 ± 181 (8)^e^	69 ± 52 (12)	59 ± 47 (6)	56 ± 41 (2)
PM_4 _Smoker	275 ± 195 (2)	182 ± 97 (4)	159 ± 88 (4)	164 ± 54 (4)	103 ± 8 (2)	150 ± 81 (2)
OC Non smoker	43 ± 12 (12)	34 ± 7 (6)	43 ± 12 (8)	48 ± 16 (12)	35 ± 14 (6)	37 ± 1 (2)
OC Smoker	85 ± 31 (2)	107 ± 71 (4)	137 ± 63 (4)	97 ± 34 (4)	68 ± 8 (2)	95 ± 55 (2)
EC Non smoker	11 ± 3 (12)	7 ± 2 (6)	5 ± 3 (8)	7 ± 2 (12)	7 ± 3 (6)	2 ± 1 (2)
EC Smoker	14 ± 4 (2)	13 ± 13 (4)	11 ± 5 (4)	10 ± 3 (4)	9 ± 1 (2)	7 ± 6 (2)

^a^: Measurements done during winter time

^b^: Results are mean ± SD (n = 2)

^c^: n.a: not available

^d^: Results are mean ± SD (n); units in μg/m^3^

^e^: including a heavily exposed worker (570 μg/m^3^)

For non-smokers, personal PM_4 _and OC air concentrations were always higher than the corresponding stationary air concentrations (Table 2). As expected, smokers presented higher exposure to PM_4 _and OC compared to non-smokers (Table 2).

### Urinary biomarkers of PAH exposure

**Figure 1:**
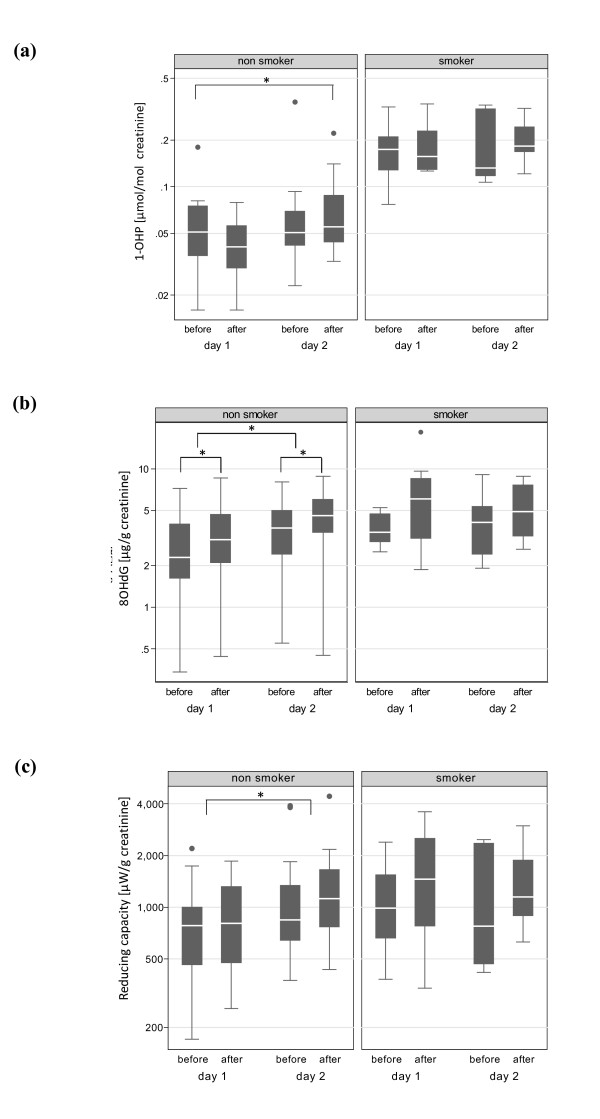
**Levels of 1-OHP, 8OHdG and reducing species in urine**. Concentrations of 1-OHP (a), 8OHdG (b) and reducing capacity (c) in urine samples of workers, presented as a function of their smoking status and time of sampling. Concentrations are expressed as μmol/mol creatinine for 1-OHP, μg/g creatinine for 8OHdG, and μW/g creatinine for the reduced species. Horizontal line in the box plot indicates the median, with 25 and 75% of the values being inside the box. Whiskers correspond to 95% of all the values, and dots to outliers. * indicate a statistically significant difference (p < 0.05).

Figure 1(a) shows the urinary 1-OHP levels during the two consecutive days of work. A clear difference was observed between non-smokers (0.06 ± 0.04 μmol/mol creatinine, average value for both days, n = 94) and smokers (0.19 ± 0.08 μmol/mol creatinine, average value for both days, n = 31). The linear mixed model (see Additional file 1 Table S3) confirmed the effect of smoking (p < 0.001), and identified a seasonal effect (p = 0.02), and a trend for self-reported exposure during the week-end (p = 0.08), which could be attributable to exposure to barbecue activities during the summer. A significant difference existed for non-smokers between urinary concentrations at the beginning of day 1 and those at the end of day 2 (p = 0.006).

### Urinary levels of 8OHdG

The urinary concentrations of 8OHdG during both days are shown in Figure 1(b), and the associated statistics in Table 3. The model was shown not to be influenced by night shift, BMI, season, whereas current smoking and self-reported respiratory problems were partially associated with 8OHdG. Independent of exposure (Model A1 of Table 3), urinary levels of 8OHdG were 40% higher for smokers than for non-smokers, but this difference was not statistically significant (p = 0.175). Statistically significant differences were observed between beginning and end of shifts (32% difference, p < 0.001) and between the two days among non-smokers (40% difference, p < 0.001). No increase between days was observed for smokers. PM_4 _levels had no statistical influence on the urinary 8OHdG levels but this biomarker was significantly influenced by OC and NO_x _(both with random effect models-Table 3 and fixed models - Additional file 1 Table S6), and particulate copper content (only for the random effect models-Table 3). When these three variables were fitted simultaneously with the random effect model, none was found to be significant. Non-parametric correlation tests between these three exposure variables indicated that OC and NO_x _were significantly correlated. In contrast to the above findings for stationary exposure variables, the personal exposure to PM_4_, OC and EC were not significantly correlated to 8OHdG during these two days (see Additional file 1 Table S4 for the random effect models).

**Table 3:** Coefficients with standard error and p-value for the different mixed models used for explaining the time trend of urinary 8OHdG (log corrected).

	Smoker	Respiratory problems	Between-day^a^	Within day	Constant	OC	NOx	Cu
**Model A1: No exposure**								
Coefficient	0.27 ± 0.20	0.63 ± 0.33	0.33 ± 0.07	0.25 ± 0.06	0.75 ± 0.2	-	-	-
p	0.175	0.055	< 0.001	< 0.001	< 0.001	-	-	-
**Model A2: including stationary OC**								
Coefficient	0.34 ± 0.20	0.68 ± 0.33	0.29 ± 0.07	-0.47 ± 0.27	1.22 ± 0.3	0.03 ± 0.01	-	-
p	0.087	0.039	< 0.001	0.083	< 0.001	0.007	-	-
**Model A3: including stationary NOx**								
Coefficient	0.46 ± 0.23	0.67 ± 0.34	0.39 ± 0.08	-0.22 ± 0.19	0.90 ± 0.3	-	7.7.10^-4^ ± 2.7. 10^-4^	-
p	0.05	0.052	< 0.001	0.259	< 0.001	-	0.004	-
**Model A4: including stationary Cu**								
Coefficient	0.36 ± 0.20	0.61 ± 0.31	0.36 ± 0.07	0.12 ± 0.09	0.60 ± 0.2	-	-	1.5.10^-3 ^± 0.7. 10^-3^
p	0.069	0.047	< 0.001	0.150	0.001	-	-	0.029

^a^: restricted to non-smokers

### Urinary levels of the reducing species

The urinary concentration of reducing species during the two sampling days is shown in Figure 1(c). As for 8OHdG, the levels of excreted reducing species were 35% higher among smokers (p = 0.08) compared to non-smokers, and 41% higher for workers with self-reported respiratory diseases (p = 0.08, see Table 4). Adjusted for these factors, the level of reducing species increased by 14% (p = 0.06) within the shifts, although this increase seemed to be restricted to day 2. Again a significant overall between-day increase was observed only among non-smokers (p = 0.002). None of the air concentrations (stationary - Table 4 and personal - Additional file 1 Table S5) had any significant association to the within-shift urinary levels of reducing species. This result indicated that the measured reducing capacity in urine was not directly influenced by the different exposure variables.

**Table 4:** Coefficients with standard error and p-value for the different mixed models explaining the time trend of the urinary concentrations of water-soluble reduced species (log corrected).

	Smoker	Respiratory problems	Between-day^a^	Within day	Constant	OC	NOx	Cu
**Model A1: No exposure**								
Coefficient	0.30 ± 0.17	0.35 ± 0.20	0.35 ± 0.10	0.17 ± 0.09	6.5 ± 0.11	-	-	-
p	0.081	0.080	0.001	0.060	< 0.001	-	-	-
**Model A2: including stationary OC**								
Coefficient	0.31 ± 0.18	0.39 ± 0.21	0.33 ± 0.10	-0.22 ± 0.38	6.7 ± 0.41	0.01 ± 0.01	-	-
p	0.082	0.060	0.002	0.563	< 0.001	0.293	-	-
**Model A3: including stationary NOx**								
Coefficient	0.40 ± 0.20	0.37 ± 0.22	0.37 ± 0.11	-0.14 ± 0.26	6.6 ± 0.28	-	5.8.10^-4 ^± 3.6. 10^-4^	-
p	0.044	0.093	0.001	0.596	< 0.001	-	0.112	-
**Model A4: including stationary Cu**								
Coefficient	0.36 ± 0.18	0.34 ± 0.20	0.38 ± 0.10	0.04 ± 0.11	6.4 ± 0.18	-	-	1.5.10^-3 ^± 0.9. 10^-3^
p	0.045	0.090	< 0.001	0.760	< 0.001	-	-	0.093

^a^: restricted to non-smokers

### Correlation between urinary 8OHdG and reducing capacity

**Figure 2:**
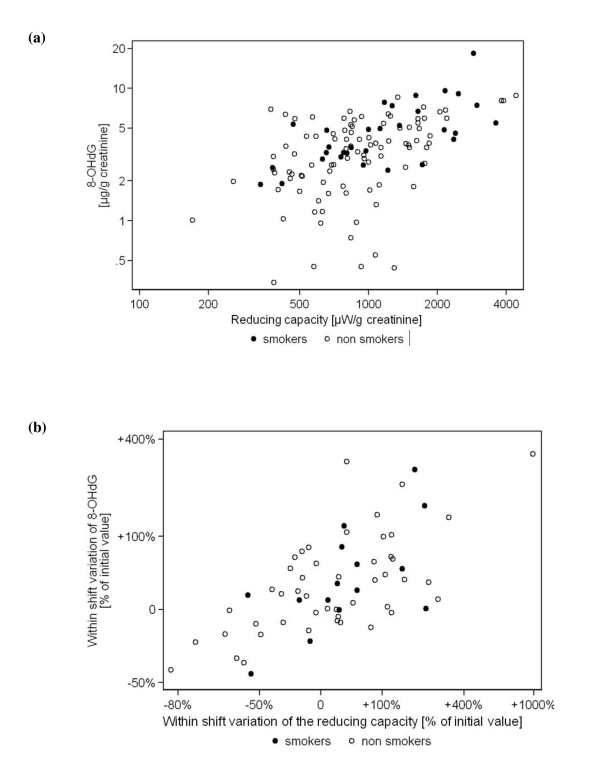
**Correlation between 8OHdG and reducing species**. (a) Correlation between urinary levels of 8OHdG (in μg/g creatinine) and reduced species (in μW/g creatinine) for all collected samples. (b) Correlation between within-shift variation of 8OHdG (% of initial value) and within-shift reduced species (% of initial value) for smokers and non smokers.

A statistically significant correlation (Spearman rho = 0.53, p < 0.0001) was observed between urinary levels of log-transformed 8OHdG and reducing capacity for all workers (smokers and non-smokers, Figure 2(a)). Further analysis revealed that the within-shift variation of log-transformed 8OHdG concentration was also correlated with the within-shift variation of the reducing species (Spearman ρ = 0.59, p < 0.0001; Figure 2(b)). The range of variation for reducing species (-80% to +1000%) was much greater than that of 8OHdG (-50% to +400%). Both of these values indicate that a tight association is present between urinary 8OHdG considered as a marker of oxidative stress and the amount of excreted reducing species.

## Discussion

This study shows that exposure to low concentrations of PM_4 _and related combustion-derived compounds was associated to an increase in urinary 8OHdG levels during two consecutive days in non-smoking male bus mechanics. This increase in oxidative stress markers was associated with increased urinary level of water soluble reducing species.

The quality of a panel study depends strongly on the exposure characterization [19]. In this work, an important effort was spent to characterize it as thoroughly as possible. The low occupational exposure to PM_4 _in the present study is comparable to two other studies for similar workplaces [39,40]. We noticed that the PM_4 _concentrations were lower during night time, possibly due to reduced work activities. OC concentrations were comparable to those obtained in previous studies conducted in bus depots [40,41]. The presence of secondary organic aerosol is suggested by the elevated proportion of OC relative to EC. EC, a primary pollutant emitted during incomplete combustion of fossil and carbonaceous fuels, is often used as a surrogate for diesel particles. Approximately 75% of a typical diesel particle is EC, depending on engine operating conditions [42]. The EC contribution to total PM_4 _was between 12 and 24% (Table 2 stationary measurements). This indicated that diesel emissions in the bus depots were not dominant. The main source of particulate matter identified at these workplaces was bus repair and maintenance. This was corroborated with the much higher personal atmospheric concentrations of PM_4 _and OC, reflecting work on engines and with organic compounds such as solvents and lubricating fluids. Moreover, the surface reactivity of the stationary collected particles in these bus depots, described in a previous paper [27], indicated that PM_4 _was quite oxidized, probably because of ageing. EC results (Table 2 stationary measurements) were comparable with those obtained in previous studies in bus depots [30,43,44]. Unlike PM_4 _and OC, the concentrations of EC measured in personal air sampling (Table 2 personal measurements) were comparable to those measured using stationary air sampling. A similar trend was observed in [40]. These results could imply that the EC concentration may be considered as rather homogeneously distributed throughout the investigated workplace. The fact that the personal exposure to PM_4 _and OC was greater than the stationary concentration was expected and is in accordance with previous studies [40,45].

We evaluated the adsorbed PAH on the collected particles because their presence may be considered a good proxy for the pro-oxidant potential of ultrafine particles [46]. The B[a]P_eq _concentration obtained in this study corresponds to urban ambient levels [47] and is in agreement with B[a]P data obtained from truck drivers [30,48]. Despite the low concentrations of B[a]P_eq _and combustion-derived particles, we detected an increase in urinary 1-OHP of non-smokers after two days of work (Figure 1(a)). This indicates that the workplace was a relevant contributor to the total PAH exposure and that metabolic processes were active. The slightly elevated 1-OHP levels observed for non-smokers on day 1 before shift compared to end of shift for the same day may be related to barbecues during the week-end. The half-life of 1-OHP in the body has been reported to be 6-35 hours [32], which suggests that the observed 1-OHP levels were mainly defined by PAH exposure of the previous 24 hours. It is known that one of the PAH activation pathways may lead to redox active quinone-like compounds, capable of oxidizing biological components [5]. However, no association was observed between log 8OHdG and log 1-OHP, neither for smokers nor for non-smokers (data not shown). This lack of correlation with log 8OHdG in non-smokers suggests that PAH did not contribute considerably as an oxidizing source in this study. Conflicting results have also been reported in the literature regarding a possible association between 8OHdG and 1-OHP. While many studies did not find any correlation [9,33,49], some reported significant correlations between these two urinary biomarkers [14,50].

The analytical determination of urinary 8OHdG is challenging, mostly due to the complexity of the matrix [19] and the use of highly specific detection techniques such as LC-MS/MS is recommended [21,51]. The urinary levels of 8OHdG determined in this study for Monday morning (0.34-7.21 μg/g creatinine; median 2.46 μg/g creatinine for non-smokers and 1.71-5.23 μg/g creatinine, median 3.36 μg/g creatinine for smokers) were in agreement with other studies reporting 8OHdG concentrations in urine for controls (non-exposed non-smokers) and analyzed by HPLC techniques (3.3-5.6 μg/g creatinine, median 3.7 μg/g creatinine - [22,49,51-54]). We observed that the concentration of the oxidative stress marker 8OHdG increased over the two consecutive days of shift in non-smoking bus workers. Such an increase of urinary 8OHdG levels is in accordance with previous pre- and post-shift studies on boilermakers exposed to residual oil fly ash [9] or security guards exposed to ambient particles [55]. It is worth mentioning that contradictory results have been obtained for garage and garbage workers [49] and for workers exposed to PAH in silicon production [33], where no statistical differences could be measured between pre- and post-workshift urinary samples collected five days later. Our statistical treatment using linear mixed models suggests that the observed 8OHdG urinary increase was mostly related to workplace exposure to OC (or NO_x_) and possibly particulate copper. This result supports the hypothesis that PM components are causative for such an increase, in agreement with most of the occupational studies investigating the effect of particle exposure on 8OHdG in urine, reviewed in [25]. Particularly for copper, an association with hydroxyl radical generation potential of coarse ambient particle and the formation of 8OHdG in an acellular test has been reported [56]. The fact that PM_4 _was not associated with 8OHdG could be due to difficulties to accurately determine low particle masses under our experimental conditions.

Personal exposure characterization is reported to be more strongly associated with the 8OHdG in lymphocytes than for stationary monitoring stations [57]. Surprisingly, we found only correlations of urinary 8OHdG with stationary, but not with personal air concentrations. This could indicate that there either was a problem with the personal measurement method (for which we have no indications), or that the stationary measurements at the workplace were a better representation of the hazard-relevant particles. In our study, personal concentrations are thought to be strongly influenced by newly emitted compounds, as volunteers are working near the particle sources. It is known that diesel particles possess an intrinsic ability to act as oxidant [58] and differences in the chemical composition of PM are important for the induction of DNA damage [59]. Based on a recent study indicating that aged diesel particles present a higher oxidant generation and potential toxicity than fresh ones [60], we speculate that the stationary concentrations represent somewhat aged particles (corresponding to more oxidized particles than freshly emitted aerosols). This is supported by other measurements [27] performed at the same depots.

Reducing species like antioxidants have an important role to play in minimizing the amount of oxidative damage that may arise from the endogenous normal metabolism of oxygen or induced by exposure to exogenous reactive compounds [61]. In our study, low exposure to particle components (OC or NO_x _and Cu) led to a significant increase in urinary 8OHdG levels in non-smokers after 2 days of work (Figure 1(b)). Concomitantly, a clear association was observed between the absolute values of urinary 8OHdG and soluble reducing species (Figure 2(a)) as well as for the within-shift variations (Figure 2(b)). One possible explanation for this result seems to be that this correlation reflects a protective response of the organism to particle-induced oxidative stress. The observed increase of reducing species in urine would mirror an increased level in blood originating from a response to oxidative stress in the body monitored by the urinary 8OHdG. This explanation is in agreement with the protective tier 1 part of the hierarchical response model [7]. In the past, antioxidant responses elicited by environmental pollutants have been described [62] but results are contradictory. Increased antioxidant levels were observed in the lining fluid of volunteers after low-dose inhalation of diesel particles (approximately 100 μg/m^3 ^PM_10_) [63,64], accompanied by an increase of reduced glutathione and urate after 18 hours post-exposure. Such an increase had been attributed to an up-regulation of protective antioxidants [63]. Exposure to PM_2.5 _has also been reported to increase the serum levels of uric acid in North Carolina police officers [4]. A similar increase of plasma antioxidants in response to an increased oxidative stress was observed in newborns [22]. On the contrary, an analysis of the relationship between biomarkers of oxidative DNA damage and antioxidant status for policemen and bus drivers from three European cities [65] did not find correlations between plasma levels of vitamin A, vitamin E, vitamin C, and lymphocyte 8OHdG, while plasma vitamin C levels were negatively correlated with 8OHdG in urine of bus drivers [59]. Severe depletion of plasma antioxidants was also observed in cement plant workers, concomitantly with increased concentrations of biomarkers of lipoperoxidation [66]. High particle exposure usually associated with such activities may have overwhelmed the antioxidant control, which could explain these contradictory results. Likewise, the use of different antioxidant markers makes the comparison of these results difficult.

Another plausible explanation for the observed correlation between 8OHdG and the urinary reducing capacity could be due to changed metabolism related to circadian rhythms. Such a process would be particularly visible for the within-shift variations of these two parameters (Figure 2(b)). Indeed, the concentrations of urinary 8OHdG have been shown to increase from 6 a.m. to reach its maximum around 6 p.m. [67]. Such biological variations may contribute to the observed within-day changes of 8OHdG but not to the between-day increase. On the other side, circadian rhythms have been observed for the activity of antioxidant enzymes as well as for the synthesis of low molecular weight antioxidants (reviewed in [68]). Particularly for urate, a molecule responding to the present electrochemical measurement, a diurnal maximum in human serum (peaking at around 7 a.m.) has been reported [67]. As only 10% of urate is excreted in the urine (the remaining 90% being recirculated by the renal system [3]), an increase of this antioxidant in blood will also lead to an increase in urine. The fact that we do not observe any correlation between the reducing capacity and the exposure parameters adds some weight to the suggestion that these within-shift variations are related to endogenous processes.

The presence of confounding factors such as diet has also to be taken into account when DNA damage biomarkers are considered [19]. This parameter would have an effect only on the reducing capacity, as 8OHdG levels in urine are reported to be independent of the diet [69]. It is unlikely that the diet of the workers changed drastically during the two sampling days, suggesting that the observed urinary increase of the reducing capacity for non-smokers may be due to other influences.

## Conclusions

In summary, surveyed workers in bus depots were exposed to low levels of PM_4 _and related combustion-derived compounds. Despite this low exposure, urinary levels of 8OHdG increased significantly for non-smoking mechanics during two consecutive days of shift. This increase was correlated with the concentrations of the particle-related variables OC, NO_x_, and possibly the particulate copper content. The increase of the oxidative stress marker was accompanied by an increase of urinary levels of water soluble reducing species. This strong association is either suggestive of an increase of the effect of different protection mechanisms or could be explained by changes in the metabolism, as observed in circadian rhythms. Additional investigations should be performed in order to shed light on these issues.

## List of abbreviations used

B[a]P_eq_: Benzo[a]Pyrene equivalent concentrations; BMI: body mass index; Cu: copper; EC: elemental carbon; Fe: iron; GC-MS: gas chromatography-mass spectrometer; ISO/IEC 17025: International Organization for Standardisation, Norm for "General requirements for the competence of testing and calibration laboratories"; LC-MS/MS: liquid chromatography-tandem mass spectrometry; Mn: manganese; OC: organic carbon; 8OHdG: 8-hydroxy-2'-deoxyguanosine; 1-OHP: 1-hydroxypyrene; PAHs : polycyclic aromatic hydrocarbons; PM_4_: particles with aerodynamic diameter smaller than 4 μm; ROS: reactive oxygen species; SD: standard deviation; SPE: solid-phase extraction; μW: microwatt.

## Competing interests

The authors declare that they have no competing interests.

## Authors' contributions

**JJS**: participated in the study design and planning, was responsible for the field campaign, performed the PM measurements and characterization, evaluated and interpreted the data and participated in the writing of the manuscript. **AS**: organized the field campaign, was responsible for the field characterization of gaseous pollutants, performed the urinary 8OHdG measurements, evaluated and interpreted the data, prepared and participated in the manuscript writing. **PW**: evaluated the data, performed the statistical analysis and participated in the writing of the manuscript. **PT/GL**: performed the reducing species measurements and contributed to the writing of the manuscript. **FS**: participated in the field campaign, performed the 1-OHP analysis and contributed to the writing of the manuscript. **SD**: participated in the field campaign, performed the PAH analysis and contributed to the writing of the manuscript. **MG/MJR**: participated in the study design and contributed to the scientific content of the manuscript and its revision. **MR**: participated in the study design and planning, interpreted the toxicological data and contributed to the scientific content and manuscript revision. All authors have read and approved the final manuscript.

## Supplementary Material

Additional file 1 **Supplemental Material Manuscript ID 2422944994984550**. File giving more details about the analytical conditions for the 8OHdG determination in urine, the sampling sites as well as the results of the different statistical fixed and random models not presented in the main manuscript. Click here for file
