# Supplementary material for: Biomarkers of oxidative stress and its association with the urinary reducing capacity in bus maintenance workers
Source: J Occup Med Toxicol. 2011 May 30;6:18. doi: 10.1186/1745-6673-6-18 (PMC3135575; doi:10.1186/1745-6673-6-18)
Supplement: Additional file 1 — Supplemental Material Manuscript ID 2422944994984550. File giving more details about the analytical conditions for the 8OHdG determination in urine, the sampling sites as well as the results of the different statistical fixed and random models not presented in the main manuscript. [file 1745-6673-6-18-S1.DOCX]

**Biomarkers of oxidative stress and its association with the urinary reducing capacity in bus maintenance workers.**

Ari Setyan, Jean-Jacques Sauvain, Pascal Wild, Philippe Tacchini, Grégoire Lagger, Ferdinand Storti, Simon Deslarzes, Michel Guillemin, Michel J. Rossi, Michael Riediker

**Supplemental Material**

**Measurement of urinary 8OHdG**

The parameter settings of the LC-MS/MS are given in Table S1.

**Table S1:** Parameter settings for LC-MS/MS.

| **HPLC** | Analytical column | Polaris C-18 (L = 50 mm, ID = 2mm, 5 µm) |
| --- | --- | --- |
|  | Program mobile phase^a^ | 0’ = 95% A / 5% B  3’ = 95% A / 5% B  5’ = 30% A / 70% B  10’ = 30% A / 70% B  10.1’ = 95% A / 5% B  15’ = 95% A / 5% B |
|  | Flow rate | 0.3 ml/min |
|  | Column temperature | 40 °C |
|  | Injection volume | 20 µl |
| **MS/MS** | Ionization mode | Negative |
|  | Gas | Air |
|  | Housing temperature | 65 °C |
|  | Nebulization temperature | 290 °C |
|  | Drying gas temperature | 250 °C |
|  | Needle tension | - 4500 V |
|  | Shield tension | - 600 V |
|  | Capillary tension | - 68 V |
|  | Ion monitoring in 1^st^ quadrupole | m/z 282 |
|  | Argon collision energy in 2^nd^ quadrupole | - 19 V for m/z = 282 |
|  | Ion monitoring in 3^rd^ quadrupole | m/z 192 |

^a^ solution A = 0.1% formic acid in water (pH 2.7); solution B = 0.1% formic acid in methanol.

**Site description**

A rough description of each sampling location, including the date, average temperature and relative humidity during the sampling shift is given in Table S2.

**Table S2.** Description of the sampling sites, including the average temperature and humidity during the sampling period.

| **Sampling site** | **Description of the sampling sites** | **Sampling date** | **Average**  **temperature [°C]** | **Average relative humidity [% rH]** |
| --- | --- | --- | --- | --- |
| Bus depot 1  summer day | Mechanical yard: repair and maintenance of buses and trolleybus | 27.03.2006  day | 22.2 ± 1.7 | 33.5 ± 2.9 |
|  |  | 28.03.2006  day | 20.8 ± 0.8 | 42.7 ± 3.8 |
| Bus depot 2  summer day | Mechanical yard: repair and maintenance of buses | 22.05.2006  day | 23.2 ± 1.7 | 42.9 ± 3.6 |
|  |  | 23.05.2006  day | 22.6 ± 1.2 | 36.4 ± 7.6 |
| Bus depot 2  summer night | Nearby a track used by all the buses and trams to join their respective parking place | 22.05.2006  night | 23.2 ± 0.7 | 53.3 ± 5.8 |
|  |  | 23.05.2006  night | 21.4 ± 0.6 | 34.7 ± 3.6 |
| Bus depot 2  winter day | Mechanical yard: repair and maintenance of buses | 12.02.2007  day | 21.0 ± 0.2 | 40.2 ± 3.6 |
|  |  | 13.02.2007  day | 20.8 ± 0.6 | 34.7 ± 2.9 |
| Bus depot 3  summer day | Mechanical yard: repair and maintenance of buses and trolleybus | 06.06.2006  day | 24.9 ± 1.8 | 29.9 ± 7.3 |
|  |  | 07.06.2006  day | 24.1 ± 1.2 | 26.4 ± 5.1 |
| Bus depot 3  summer night | Maintenance yard: cleaning and fuel filling of buses and trolleybus | 06.06.2006  night | 21.3 ± 1.9 | 43.7 ± 8.2 |
|  |  | 07.06.2006  night | 19.5 ± 2.5 | 33.8 ± 11.5 |

**Mixed models for 1-OHP**

The results of the mixed model explaining the time trend of urinary log(1-OHP) are given in Table S3.

**Table S3.** Coefficients with standard error and p value for the mixed models explaining the time trend of urinary log(1-OHP).

|  | **No exposure** | |
| --- | --- | --- |
| **log(1-OHP)** | **Coefficient** | **p** |
| **Smoker** | 1.17±0.15 | <0.001 |
| **Week-end exposure** | 0.35±0.20 | 0.080 |
| **Winter** | -0.20±0.09 | 0.023 |
| **Shift 1- shift 2**  **Shift 1- shift 3**  **Shift 1- shift 4** | -0.10±0.10  0.09±0.10  0.26±0.09 | 0.289  0.354  0.006 |
| **Constant** | -2.91±0.11 | 0.000 |

**Mixed models with personal exposure**

The results of the mixed model tested to explain the time trend of urinary log(8OHdG) are given in Table S4, whereas the one used for log(reducing capacity) are given in Table S5.

**Table S4.** Mixed model for the relationship between log 8OHdG and personal exposure.

|  | **Smoker** | **Respiratory problems** | **Between-day^a^** | **Within day** | **Constant** | **Personal PM_4_** | **Personal OC** | **Personal EC** |
| --- | --- | --- | --- | --- | --- | --- | --- | --- |
| **Model with no exposure** | | | | | | | | |
| Coefficient | 0.27±0.20 | 0.63±0.33 | 0.33±0.07 | 0.25±0.06 | 0.75±0.2 | - | - | - |
| p | 0.175 | 0.055 | <0.001 | <0.001 | <0.001 | - | - | - |
| **Model including personal PM_4_** | | | | | | | | |
| Coefficient | 0.28±0.21 | 0.63±0.34 | 0.32±0.07 | 0.27±0.09 | 0.48±0.2 | -1.9.10^-4^ ±5.5.10^-4^ | - | - |
| p | 0.174 | 0.062 | <0.001 | 0.002 | 0.017 | 0.732 | - | - |
| **Model including personal OC** | | | | | | | | |
| Coefficient | 0.26±0.21 | 0.64±0.34 | 0.33±0.07 | 0.24±0.10 | 0.52±0.2 | - | 2.2.10^-4^ ±1.4.10^-3^ | - |
| p | 0.214 | 0.060 | <0.001 | 0.023 | 0.013 | - | 0.875 | - |
| **Model including personal EC** | | | | | | | | |
| Coefficient | 0.33±0.20 | 0.63±0.34 | 0.32±0.07 | 0.43±0.11 | 0.31±0.2 | - | - | -2.0.10^-2^ ±1.1.10^-2^ |
| p | 0.107 | 0.061 | <0.001 | <0.001 | 0.141 | - | - | 0.056 |

^a^: restricted to non-smokers

**Table S5.** Mixed model for the relationship between log (reducing capacity) and personal exposure.

|  | **Smoker** | **Respiratory problems** | **Between-day^a^** | **Within day** | **Constant** | **Personal PM_4_** | **Personal OC** | **Personal EC** |  |
| --- | --- | --- | --- | --- | --- | --- | --- | --- | --- |
| **Model with no exposure** | | | | | | | | |  |
| Coefficient | 0.30±0.17 | 0.35±0.20 | 0.35±0.10 | 0.17±0.09 | 6.5±0.11 | - | - | - |  |
| p | 0.081 | 0.080 | 0.001 | 0.060 | <0.001 | - | - | - |  |
| **Model including personal PM_4_** | | | | | | | | |  |
| Coefficient | 0.33±0.18 | 0.35±0.21 | 0.34±0.10 | 0.25±0.13 | 6.24±0.2 | -6.8.10^-4^ ±7.6.10^-4^ | - | - |  |
| p | 0.070 | 0.091 | 0.001 | 0.049 | <0.001 | 0.372 | - | - |  |
| **Model including personal OC** | | | | | | | | | |
| Coefficient | 0.34±0.19 | 0.34±0.20 | 0.35±0.10 | 0.23±0.15 | 6.26±0.21 | - | -1.1.10^-3^ ±1.9.10^-3^ | - |  |
| p | 0.068 | 0.095 | 0.001 | 0.110 | <0.001 | - | 0.573 | - |  |
| **Model including personal EC** | | | | | | | | | |
| Coefficient | 0.32±0.18 | 0.35±0.20 | 0.34±0.11 | 0.28±0.16 | 6.22±0.21 | - | - | -1.2.10^-3^ ±1.5.10^-3^ |  |
| p | 0.067 | 0.080 | 0.001 | 0.079 | <0.001 | - | - | 0.417 |  |

^a^: restricted to non-smokers

**Fixed models with stationary exposure variables**

The results of the fixed models tested to explain the time trend of urinary log (8OHdG) are given in Table S6, whereas the one used for log (reducing capacity) are given in Table S7.

**Table S6.** Fixed model explaining the time trend for log (8OHdG).

|  | **Between-day^a^** | **Within day** | **Constant** | **OC** | **NOx** | **Cu** |
| --- | --- | --- | --- | --- | --- | --- |
| **Model with no exposure** | | | | | | |
| Coefficient | 0.32±0.07 | 0.25±0.06 | 0.94±0.05 | - | - | - |
| p | <0.001 | <0.001 | 0.001 | - | - | - |
| **Model including stationary OC** | | | | | | |
| Coefficient | 0.28±0.07 | -0.39±0.28 | 1.34±0.29 | 0.02±0.01 | - | - |
| p | <0.001 | 0.165 | <0.001 | 0.020 | - | - |
| **Model including stationary NOx** | | | | | | |
| Coefficient | 0.37±0.08 | -0.16±0.20 | 1.09±0.21 | - | 6.7.10^-4^  ±2.7. 10^-4^ | - |
| p | <0.001 | 0.412 | <0.001 | - | 0.017 | - |
| **Model including stationary Cu** | | | | | | |
| Coefficient | 0.34±0.07 | 0.15±0.09 | 0.78±0.12 | - | - | 1.1.10^-3^ ±0.7. 10^-3^ |
| p | <0.001 | 0.073 | <0.001 | - | - | 0.100 |

^a^: restricted to non-smokers

**Table S7.** Fixed model explaining the time trend for log (reducing capacity).

|  | **Between-day^a^** | **Within day** | **Constant** | **OC** | **NOx** | **Cu** |
| --- | --- | --- | --- | --- | --- | --- |
| **Model with no exposure** | | | | | | |
| Coefficient | 0.34±0.10 | 0.17±0.09 | 6.63±0.08 | - | - | - |
| p | 0.001 | 0.067 | <0.001 | - | - | - |
| **Model including stationary OC** | | | | | | |
| Coefficient | 0.32±0.10 | -0.24±0.40 | 6.88±0.43 | 0.01±0.01 | - | - |
| p | 0.003 | 0.558 | <0.001 | 0.306 | - | - |
| **Model including stationary NOx** | | | | | | |
| Coefficient | 0.37±0.11 | -0.21±0.28 | 6.84±0.30 | - | 6.5.10^-4^  ±3.9. 10^-4^ | - |
| p | 0.002 | 0.459 | <0.001 | - | 0.098 | - |
| **Model including stationary Cu** | | | | | | |
| Coefficient | 0.36±0.10 | 0.06±0.12 | 6.56±0.17 | - | - | 1.3.10^-3^ ±1.0. 10^-3^ |
| p | 0.001 | 0.624 | <0.001 | - | - | 0.190 |

^a^: restricted to non-smokers
